# Supplementary material for: Design and degradation of permanently porous vitamin C and zinc-based metal-organic framework
Source: Commun Chem. 2022 Feb 25;5:24. doi: 10.1038/s42004-022-00639-x (PMC9814379; doi:10.1038/s42004-022-00639-x)
Supplement: Supplementary file 1 — Supplementary Information [file 42004_2022_639_MOESM1_ESM.pdf]

Supplementary information

Design and degradation of permanently porous vitamin C and zinc-based metal-organic framework

Tia K. Tajnšek[1,2], Erik S. Grape[3], Tom Willhammar[3], Tatjana A. Jelić[4], Uroš Javornik[1], Goran Dražić[1], Nataša Z. Logar[1,5], Matjaž Mazaj[1]*

[1] National Institute of Chemistry, Hajdrihova 19, 1000 Ljubljana, Slovenia

[2] Faculty of Inorganic Chemistry and Technology, University of Ljubljana, Večna pot 113, 1000 Ljubljana, Slovenia

[3] Stockholm University, Frescativägen 8, 106 91 Stockholm, Sweden

[4] Ruđer Bošković Institute, Bijenička cesta 54, 1000 Zagreb, Croatia

[5] University of Nova Gorica, Vipavska 13, 5000 Nova Gorica, Slovenia

Corresponding author: Matjaž Mazaj, matjaz.mazaj@ki.si

Table of Contents

1 Supplementary Methods 3

1.1 Synthetic approaches 3

1.2 Characterization methods 5

1.2.1 Crystal structure analysis 6

1.2.2 Porosity properties analysis 12

1.2.3 Acid metal sites in activated bioNICS-1 material 14

1.2.4 Thermal properties 15

2 Supplementary Note 1: Structure stability in aqueous media 16

2.1 The aqueous stability test. 16

2.2 Liquid NMR 21

3 Supplementary Note 2: Kinetics and mechanism of structure degradation 23

3.1 Zn(II) release kinetics. 23

3.2 Framework degradation kinetics in PBS. 23

4 Supplementary Note 3: Impregnation of bioNICS-1 with a model drug - urea 25

5 Supplentary References 28

1 Supplementary Methods

1.1 Synthetic approaches

When designing the bioNICS-1 material, the use of biocompatible or at least non-toxic solvents is required. The synthesis in water did not yield any product, most probably due to the unstable nature of ascorbic acid. It readily oxidizes into dehydroascorbic acid (DHA) which is considered to be less effective as a ligand[1]. DHA–metal ion complexes are not stable and they irreversibly hydrolase to diketogulonic acid complexes of the accompanying metal cation[2]. BioNICS-1 was therefore initially prepared using EtOH as a solvent with Zn/ASC ratio of 2:1 using a conventional heating for solvothermal synthesis. Yielded nanosized crystals with the size of about 100 nm and are in aggregated form (denoted as **bioNICS-1** in further text) as can be seen from Figure S1a. In colloidal suspension they additionally agglomerate, which can be explained by near-zero charge on the surface of nanocrystals in the pH region between 4 and 7 (Figure S2). Agglomeration is a very common occurrence / problem in the world of nanoparticles[3].

With intent to synthesize larger independent crystals to aid structural analysis, the addition of acetic acid was utilized (**bioNICS-1-aa**)[4]. The optimum EtOH/acetic acid volume ratio was 10:1 (1 ml of acetic acid in 10 ml of ethanol). The presence of acetic acid in the reaction mixture indeed enhances crystal growth reaching the size up to 2 μm and inhibits agglomeration. Higher amounts of acid did not yield a crystalline product, whereas lower amounts did not make any significant improvement of crystallinity or size of the crystals in respect with the product from the initial synthesis.

BioNICS-1 material is a potential drug delivery system (DDS). Though nano-DDS do not have a strictly set size limit that would fit all administration routes, we consider a 10 – 100 nm size in at least one dimension - a size range defined by ISO[5] - a general rule of thumb[6,7]. Narrow size distribution is even more important characteristic of the nano-DDS, than nano-size range, therefore the synthesis was also done with a microwave (MW) heating source which usually results in the formation of particles uniform in size. The reaction mixture used for the conventional heating was used also for microwave heating for 1h at 120 °C (**bioNICS-1-mw**).

Activation procedure followed a simple "wash", which was done in Soxhlet apparatus in absolute EtOH, at 60°C, under constant stirring, for approximately 12h. Following the filtration, the product was dried in a vacuum oven at 100°C for 3h.

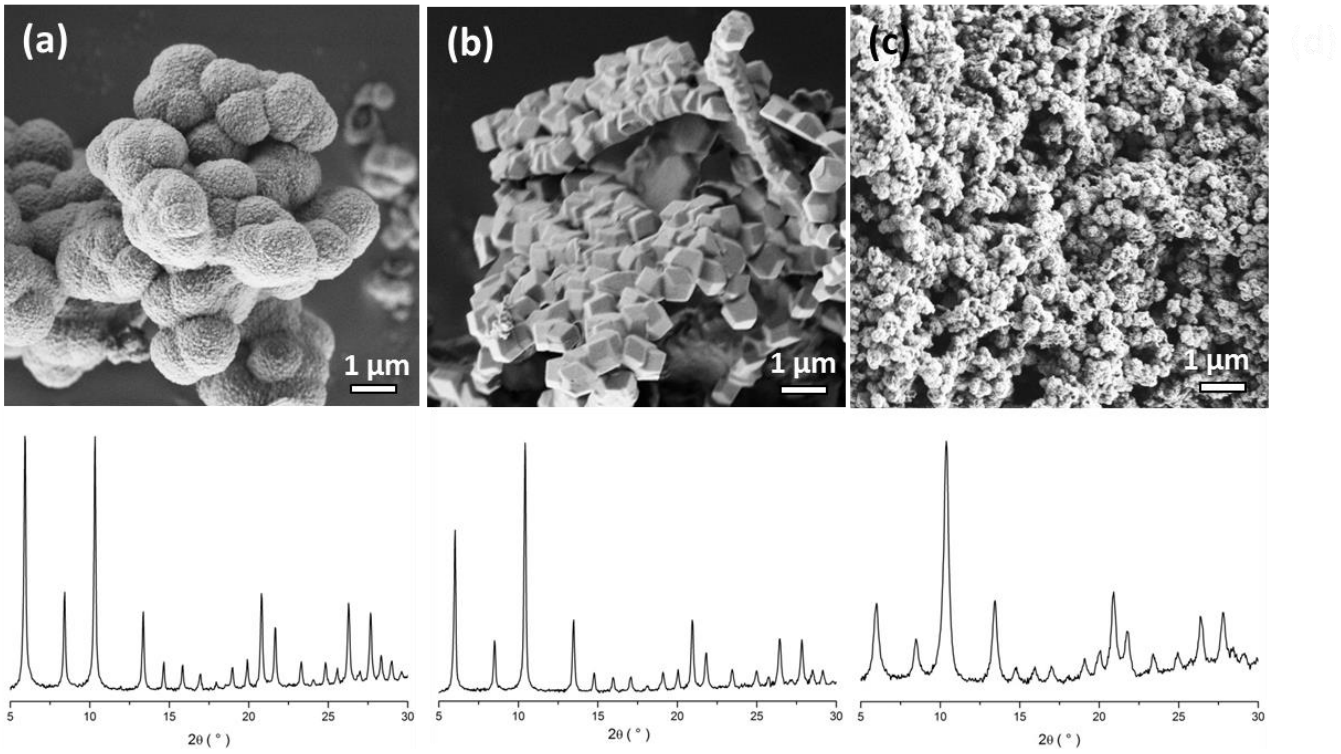

Supplementary Figure 1. SEM micrographs (above) with corresponding XRD patterns (below) of bioNICS-1 (a), bioNICS-1-aa (b) and bioNICS-1-mw (c) products.

Supplemetary Table 1. Particle size determination by indicated methods.

	**bioNICS-1 (1day)**	**bioNICS-1(aa) (acetic acid+1day)**	**bioNICS-1(mw)**	**bioNICS-1M(aa) (acetic acid + 2h)**
Scherrer	79 nm	101 nm	41 nm	91 nm
SEM	80 nm (spherical aggregates approx. 1μm)	100-120 nm (spherical aggregates with smooth surface approx. 2μm)	50 nm (spherical agglomerates approx. 300nm)	90 nm (spherical agglomerates approx. 1μm)

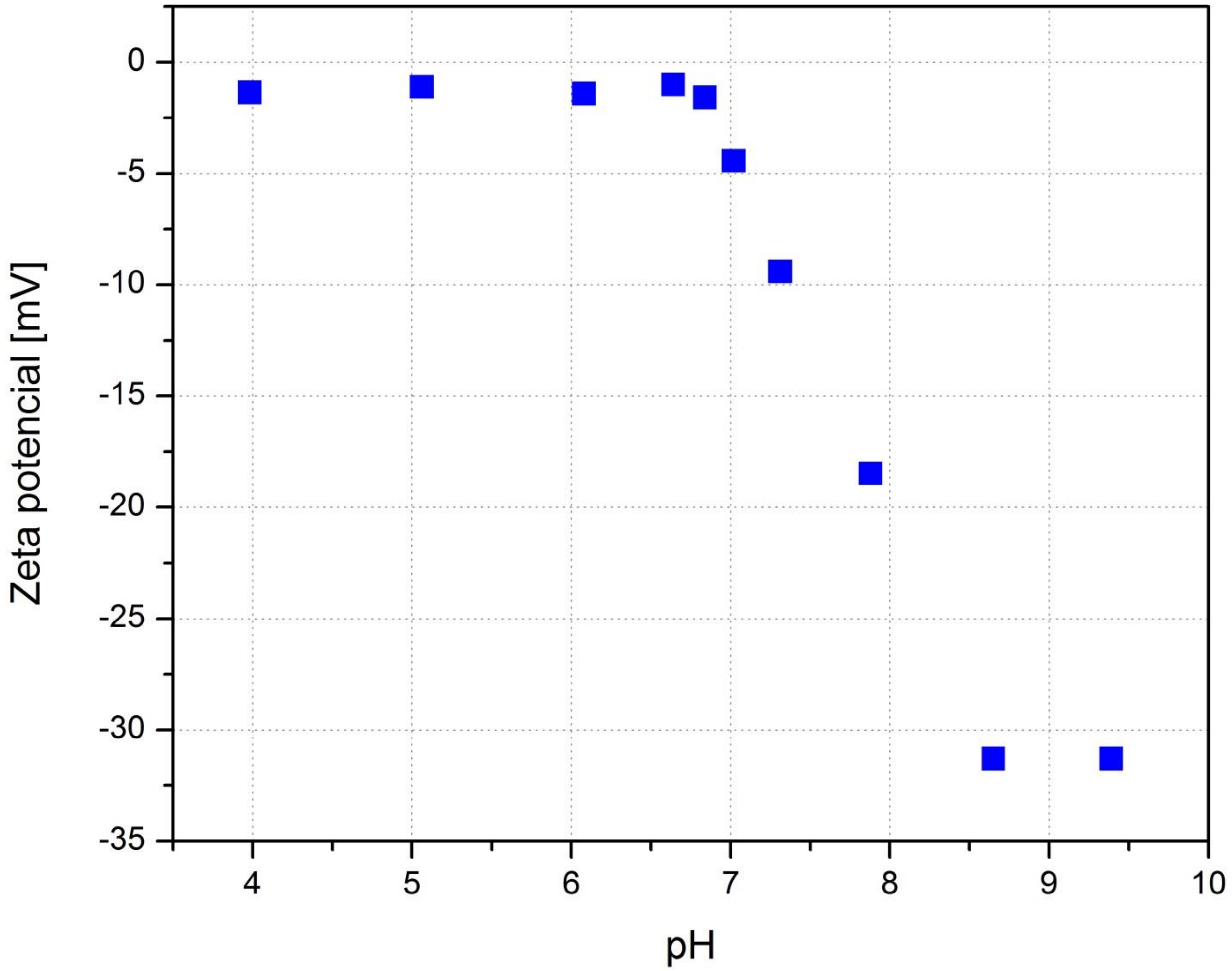

Supplementary Figure 2. Zeta potential dependence on pH of aqueous medium.

1.2 Characterization methods

X-ray powder diffraction data of the samples was collected on a PANalytical X'Pert PRO high-resolution diffractometer with CuK_a radiation (λ= 1.5406 Å) in the range from 5 to 60° (2θ) with the step of 0.034° per 100 s using fully opened 100 channel X'Celerator detector. For the purposes of Rietveld refinement of the crystal structure model, the XRD powder data was collected on the same equipment using transmission mode in the range from 5 to 90° 2θ with the step of 0.016°/300s. Prior the measurement, the bioNICS-1 sample was degassed at 150 °C overnight and sealed in glass capillary.

Morphological properties and size estimation of the samples was observed by scanning electron microscopy measurements (SEM) on Zeiss Supra™ 3VP field-emission gun (FEG) microscope. Elemental analysis was performed by energy dispersive X-ray analysis (EDAX) with an INCA Energy system attached to the above described microscope and by Perkin Elmer 2400 Series II CHNS analyser. A probe Cs-corrected Jeol ARM 200CF Scanning Transmission Electron Microscope equipped with a Centurio Energy Dispersive X-ray Spectroscopy (EDXS) system with a 100 mm^2 SDD detector was used for imaging and acquisition of STEM-EDS elemental mappings.

The thermal analysis (TG/DTG) was performed on a Q5000 IR thermogravimeter (TA Instruments, Inc.). The measurements were carried out in air flow of 10 ml/min, by heating samples from 25 °C to 700 °C at the rate of 10 °C/min. Temperature-programmed X-ray powder diffraction pattern of samples was recorded also on the PANalyticalX'Pert PRO diffractometer, additionally equipped with a high-temperature sample cell, from room temperature to 500 °C in steps of 50 °C in static air. N_2 sorption isotherms measurements were performed on Quantachrome AUTOSORB iQ3. The specific surface areas were determined by Brunauer–Emmett–Teller (BET) method based on the N_2 sorption isotherms measured at 77 K in p/p_o relative pressure range between $4 \cdot 10^{-2}$ and $6 \cdot 10^{-3}$ selected according to Roquerol plots. Before the measurement, samples were activated under vacuum at 150 °C for 15 h. Pore size distribution analysis (PSD) was performed using NLDFT procedure based on the adsorption data.

Dynamic light scattering (DLS) measurements were carried out on Zetasizer Nano ZS Malvern Instruments. Prior to the measurements, the powdered sample was dispersed in water by sonification in ultrasonic bath for 10 min. The dispersion was filtered through 0.8μm filter attached to a syringe and transferred into a polystyrene cuvette for the measurement. Data evaluation was performed using Malvern Zetasizer software. The parameters chosen for the evaluation were those for ZnO material (refractive index of 2.003 and adsorption of 0.01). The number of particles with a mean diameter was calculated.

The temperature-programmed desorption (TPD) experiments were performed using the Micromeritics AutoChem II 2920 apparatus. The sample (120 mg) was positioned inside a U-shaped quartz reactor and pre-treated in a flow Ar at 150 °C for 180 min. After pre-treatment, the latter was cooled to 50 °C and saturated with 10 % NH_3 in helium for 30 min. Weakly adsorbed NH_3 was removed in a flow of pure He for 60 min. The samples were heated to 800 °C at 20 °C/min and NH_3 desorption was monitored by a mass spectrometer (Pfeiffer Vacuum Thermostar) following the characteristic m/z fragments. Pulses of 0.5 mL 10 % NH_3 in He (Messer) were used as an external standard for NH_3-TPD signal calibration.

Zeta potential measurements were carried out on Zetasizer Nano ZS Malvern Instruments. Prior to the measurements, the powdered sample was dispersed in water by sonification in ultrasonic bath for 4 min with approximately 20% power. The dispersion was filtered through 0,8μm filter attached to a syringe and transferred into a polystyrene cuvette for the measurement. pH values of the measured suspensions were adjusted with 0.1M HCl and 0.1M NaOH solutions.

1.2.1 Crystal structure analysis

The structure of bioNICS-1 was solved from 3D electron diffraction (3DED) data collected using a JEOL JEM-2100 LaB_6 transmission electron microscope equipped with a Timepix detector (Amsterdam Scientific Instruments) while continuously tilting the crystal at 0.45° s^{-1}, during which the sample was cooled to 96 K using a Gatan 914 cryo-transfer holder. The experiment was carried out using Instamatic[8], with data reduction being performed in XDS[9]. The structure of bionics-1 was then solved using SHELXT[10], and refined using SHELXL[11] with electron scattering factors extracted from SIR2014[12]. All non-hydrogen atoms could be located in the initial structure solution from SHELXT. Upon kinematical refinement against the 3D ED data the refinement residual (R1) resulted in 0.1725. The model was further validated by refinement against PXRD data using TOPAS Academic V6[13], treating the ascorbate as a rigid body with refineable bond lengths and torsion angles.

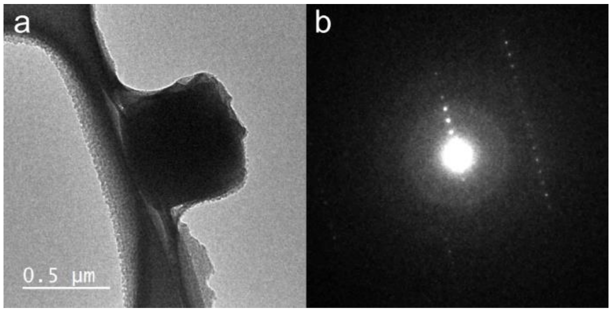

Supplementary Figure 3. a**)** The crystal used for the collection of the 3DED data and b) one of the 164 frames which were collected.

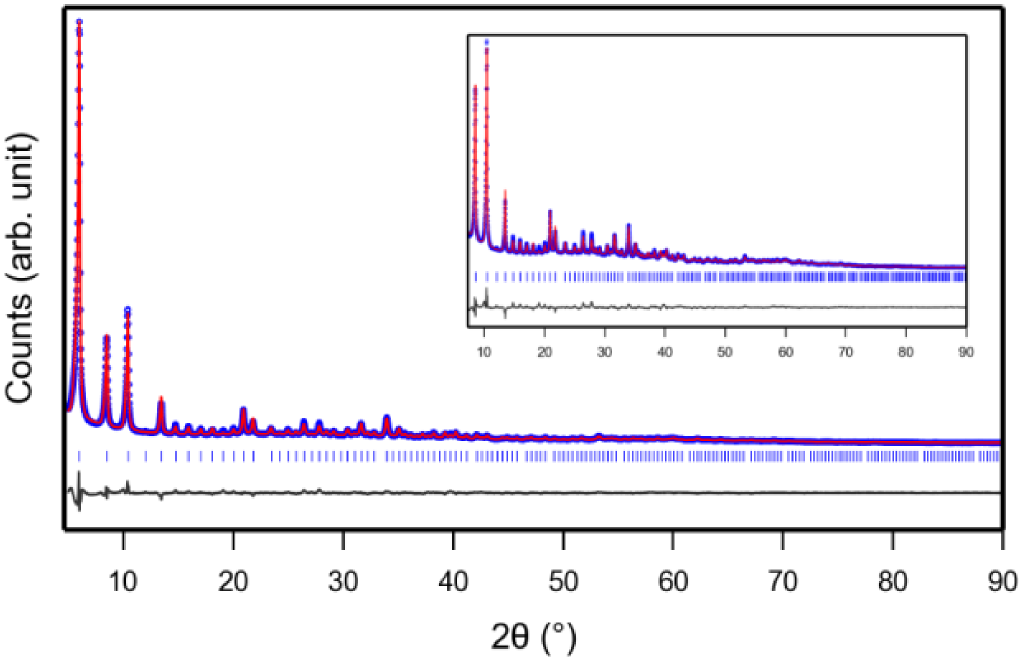

Supplementary Figure 4. Final Rietveld plot of bioNICS-1 material. Red line - experimental plot, blue dots – calculated pattern, black line - difference plot, blue tick marks – calculated reflection positions. Inset shows 2θ region with the most intense peak omitted in order to make the matching of calculated and experimental data more distinguishable.

Supplemetary Table 2. Crystal data and structure refinement using the electron diffraction data for bioNICS-1.

Empirical formula	$C_{72}O_{76}Zn_{22}$
Temperature (K)	96(2)
Wavelength	0.0251 Å
Crystal system	Cubic
Space group	$I2_13$ (No. 199)
Unit cell dimensions	a = 21.05 Å
Volume	9323 Å^3
Z	2
Rotation range	45.01° (-20.81 to 24.20°)
Index ranges	-11 ≤ h ≤ 15
	-21 ≤ k ≤ 24
	-26 ≤ l ≤ 19
Reflections collected	5108
Independent reflections	2690
	[R(int) = 0.2395]
Completeness (to 0.8 Å resolution)	93 %
R_1 (ED model) [I > 2σ(I)]	0.1725

Supplemetary Table 3. Atomic fractional positions and corresponding displacement parameters.

Atom	x	y	z	U_{iso}
Zn1	0.5202(4)	0.4798(4)	-0.0202(4)	0.010(2)
Zn2	0.8352(4)	0.4566(4)	0.0943(5)	0.010(2)
Zn3	0.75	0.3491(6)	0	0.010(2)
O1	0.5387(17)	0.5240(19)	0.0781(18)	0.036(6)
O2	0.613(19)	0.433(14)	0.001(8)	0.036(6)
O3	0.74(2)	0.40(3)	0.08(2)	0.036(6)
O4	0.628(13)	0.510(16)	0.146(10)	0.036(6)
O5	0.798(17)	0.49(5)	0.18(3)	0.036(6)
O6	0.797(16)	0.53(5)	0.05(3)	0.036(6)
O7	0.8773(18)	0.3773(18)	0.1227(18)	0.01(3)
C1	0.549(6)	0.500(9)	0.094(4)	0.036(6)
C2	0.635(13)	0.453(16)	0.056(9)	0.036(6)
C3	0.693(16)	0.44(3)	0.090(16)	0.036(6)
C4	0.690(14)	0.48(3)	0.150(14)	0.036(6)
C5	0.743(14)	0.53(4)	0.152(19)	0.036(6)
C6	0.749(15)	0.56(4)	0.088(19)	0.036(6)

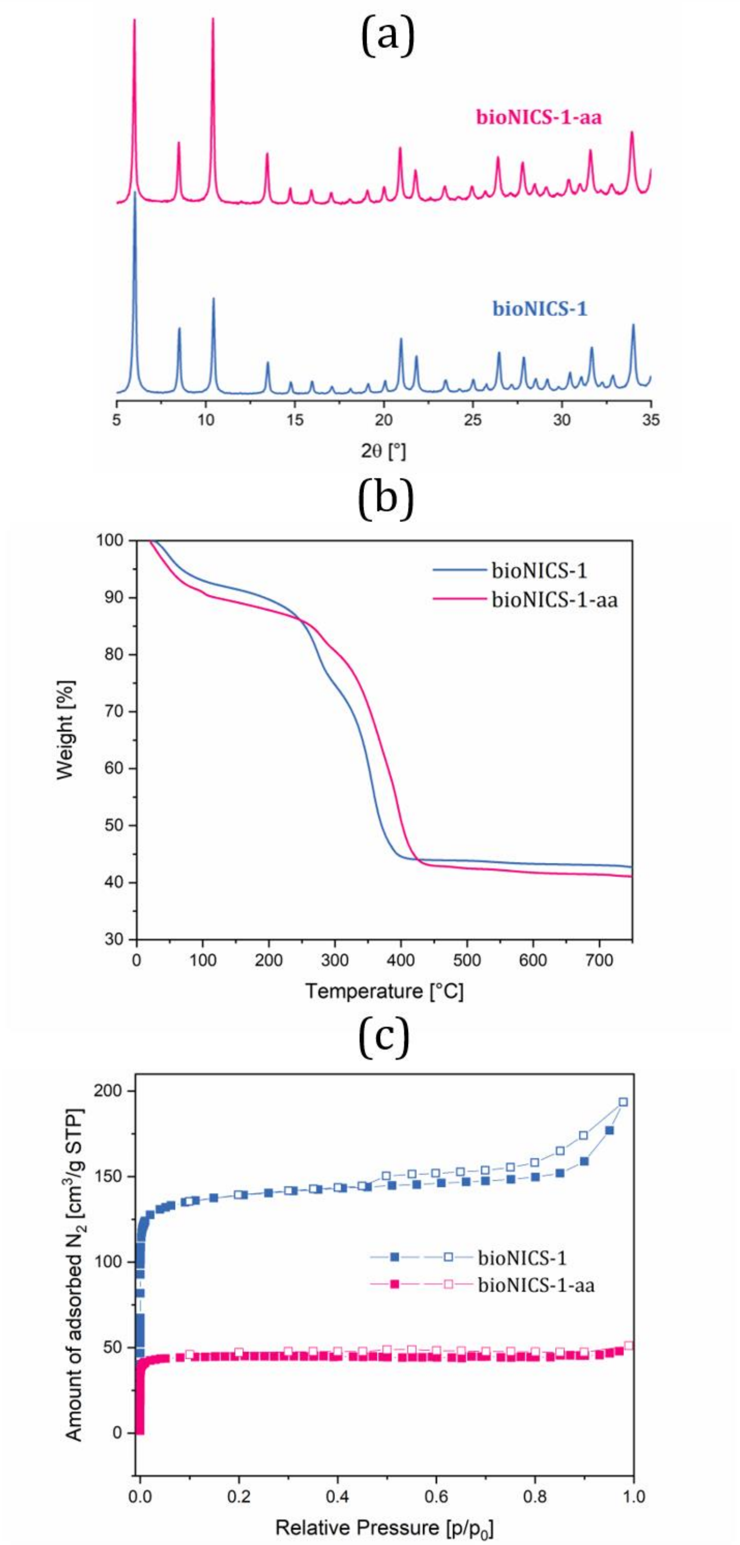

Supplementary Figure 5. comparison of (a) XRPD, (b) TGA and (c) N_2 isotherm measurements of bioNICS-1 and bioNICS1-aa product.

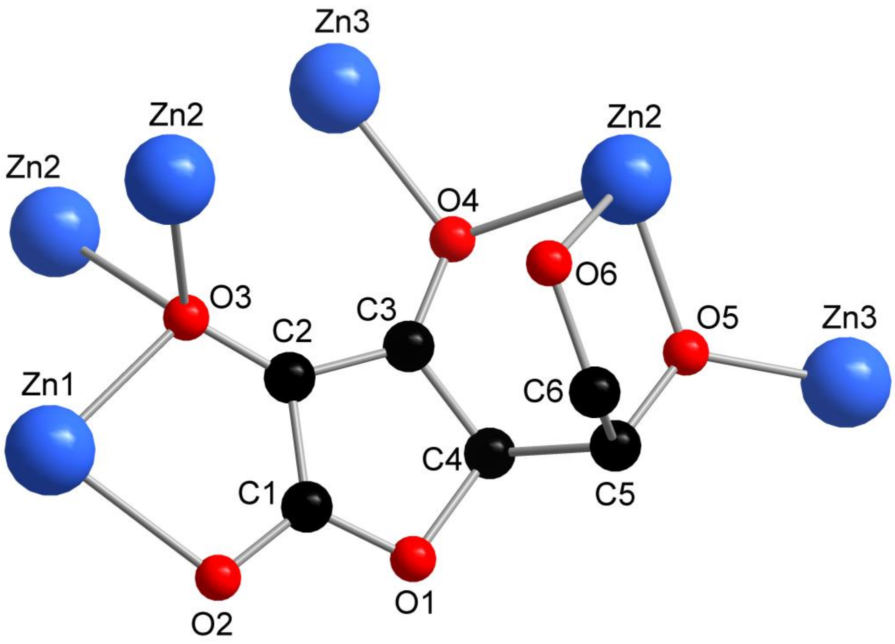

Supplementary Figure 6. Connectivity of Zn(II) centres to ascorbate ligand within bioNICS-1 structure.

1.2.2 Porosity properties analysis

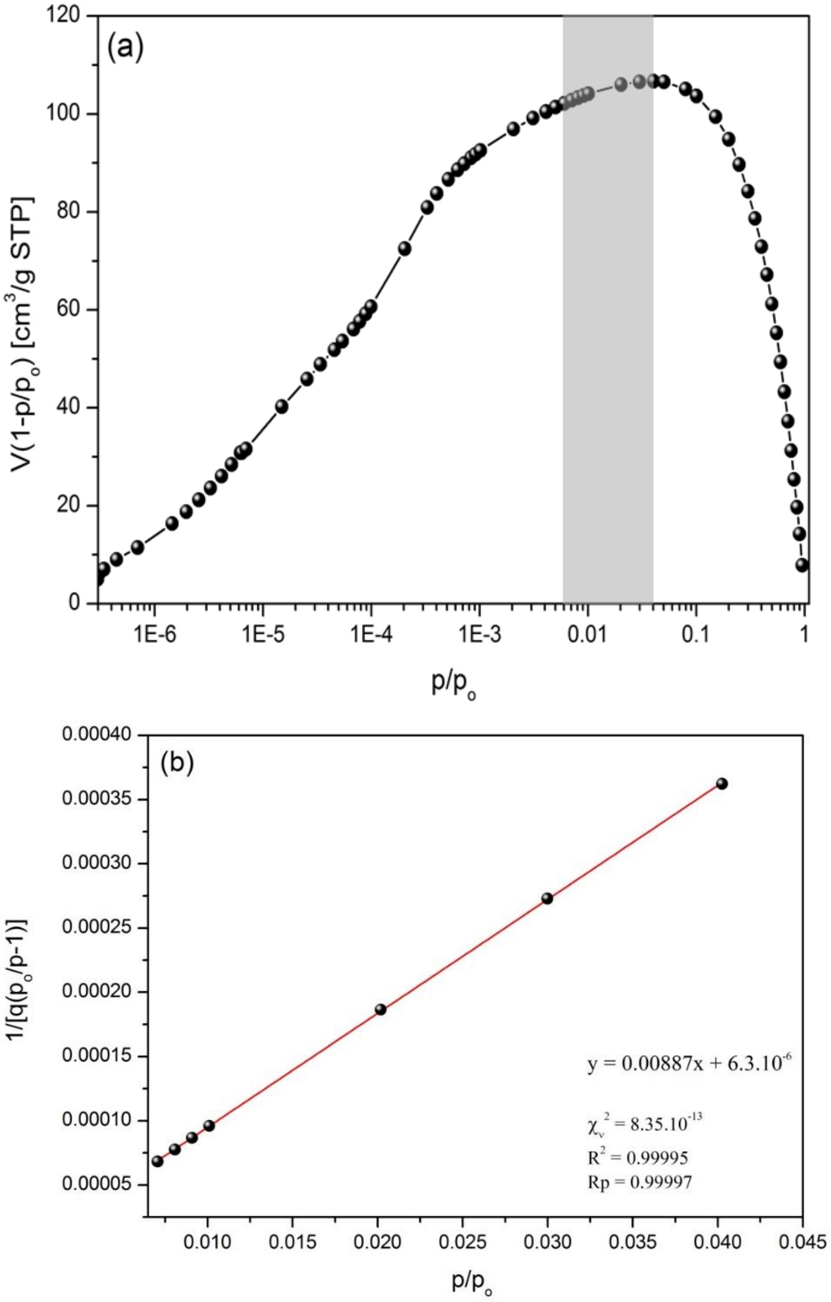

Supplementary Figure 7. (a) Rouquerol plot of the bioNICS-1 N_2 isotherm. Grey area indicates the p/p_o region considered for BET surface area calculation. (b) BET plot with statistics of the linear fit (black marks – experimental points, red line – calculated linear function). χ_v^2 – reduced chi-squared statistic, Rp – Pearson correlation coefficient.

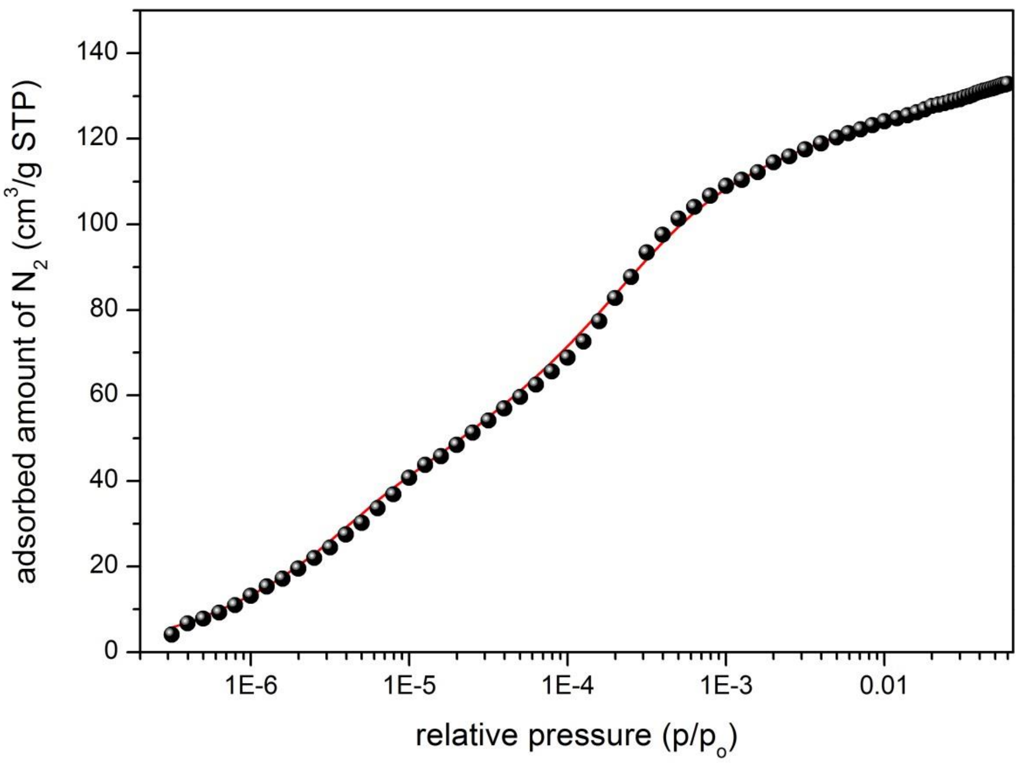

Supplementary Figure 8. N_2 adsorption isotherm of bioNICS-1 (black dots) with corresponding NLDFT fit (red line).

1.2.3 Acid metal sites in activated bioNICS-1 material

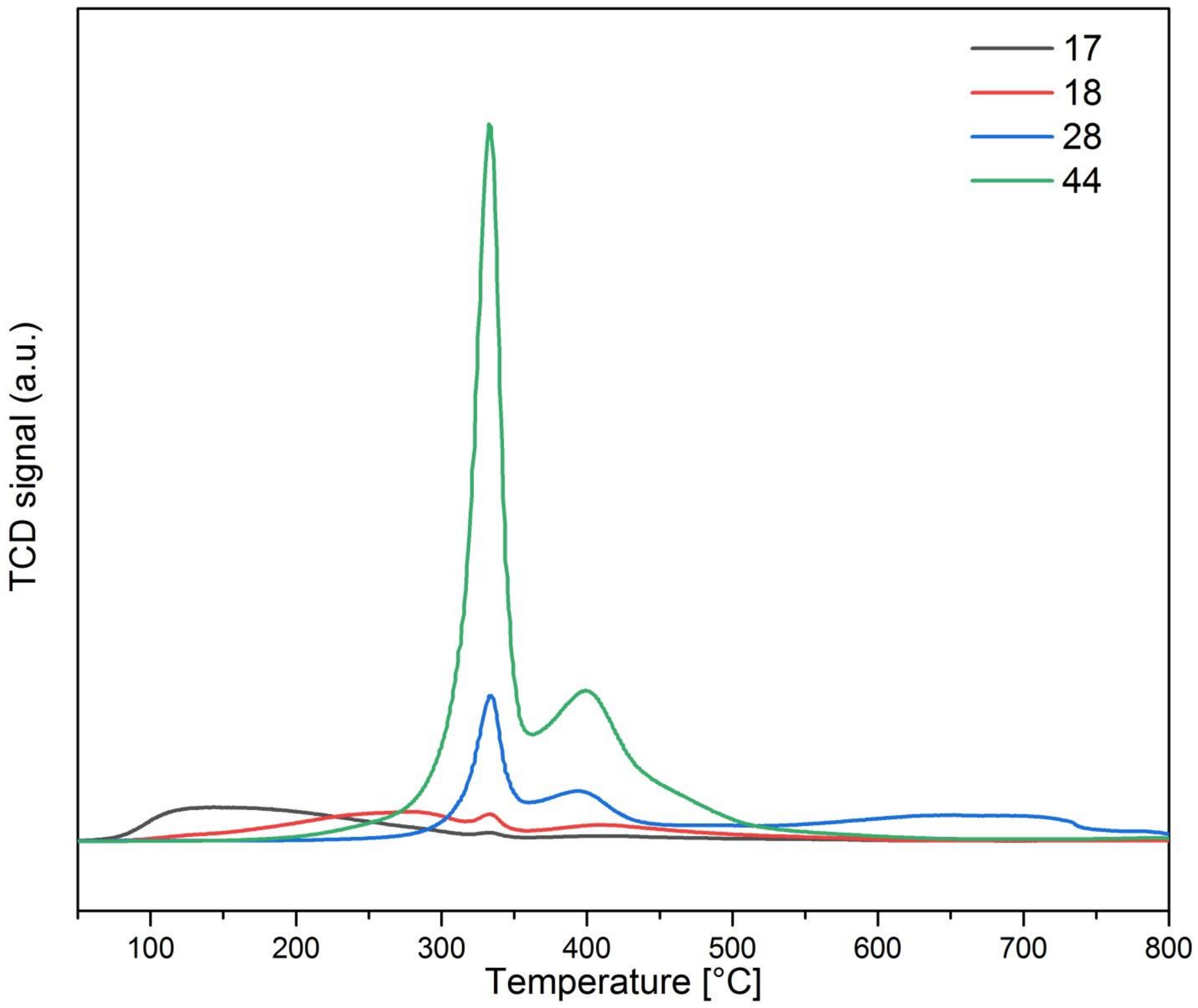

Supplementary Figure 9. TPD curves indicating desorption of different molecular species determined by MS detection of m/z fragments: ammonia (black); water (red); nitrogen (blue); carbon dioxide (green).

1.2.4 Thermal properties

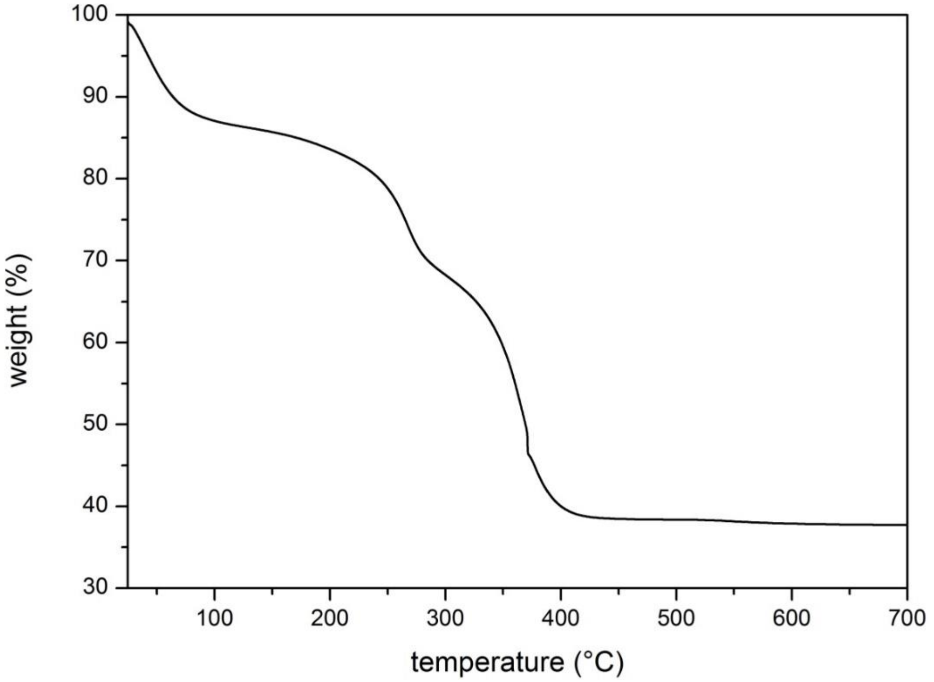

Supplementary Figure 10. Thermogravimetric curve of bioNICS-1.

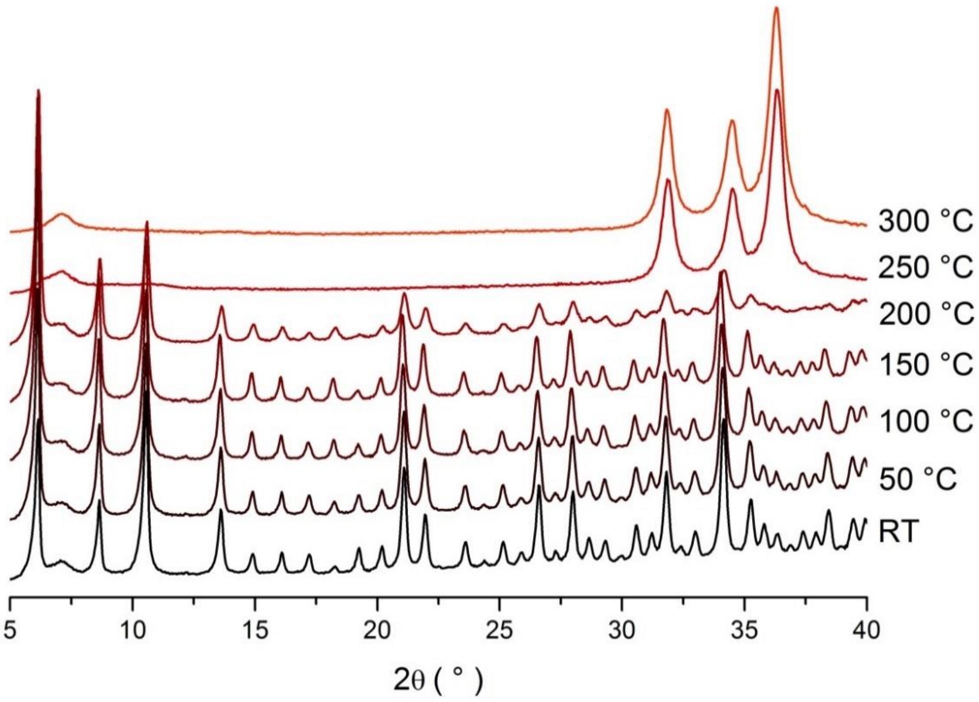

Supplementary Figure 11. Temperature-programmed powder XRD patterns of bioNICS-1 recorded at indicated temperature. Broad peak at approximately 7° 2θ is due to the Kapton foil.

2 Supplementary Note 1: Structure stability in aqueous media

2.1 The aqueous stability test.

The test was done via by slightly modified procedure presented Howarth et al.[14] Briefly, 250 mg of activated bioNICS-1 was suspended in 25 mL of purified water and saline solution, pH was regulated with HCl and NaOH to reach the values of 3.5 ; 7.4 and 9.0. Test was done in parallels; one of them was left undisturbed, the other exposed to dynamic conditions on a circular plane mixer for 24 h. Afterwards the samples were left to settle down before separately collecting the supernatant and the sediment for further analysis. The filtered sediment was left to dry on air over night and its identity was checked and confirmed with PXRD and N_2 isotherms analysis. The same procedure was repeated with phosphate buffer solution at pH 7.4.

Supernatants were collected and analysed for the presence of organic compound by NMR spectroscopy. 10 % D_2O was added to 0.5 ml of each sample. The samples were transferred to 5 mm NMR tubes and ^{1}H NMR spectra were recorded on a Bruker Avance Neo 600 MHz NMR spectrometer with a QCI cryo probe at 25 °C using excitation sculpting to suppress the water signal. Additionally ^{13}C, ^{1}H-^{13}C HSQC and ^{1}H-^{13}C HMBC NMR spectra were recorded on the sample of supernatant after treatment in phosphate buffer solution under dynamic conditions to obtain more detail about the nature of compounds in the sample. Chemical shifts were referenced externally to the chemical shift of NaTMSP (δ_H 0 ppm, δ_C 0 ppm).

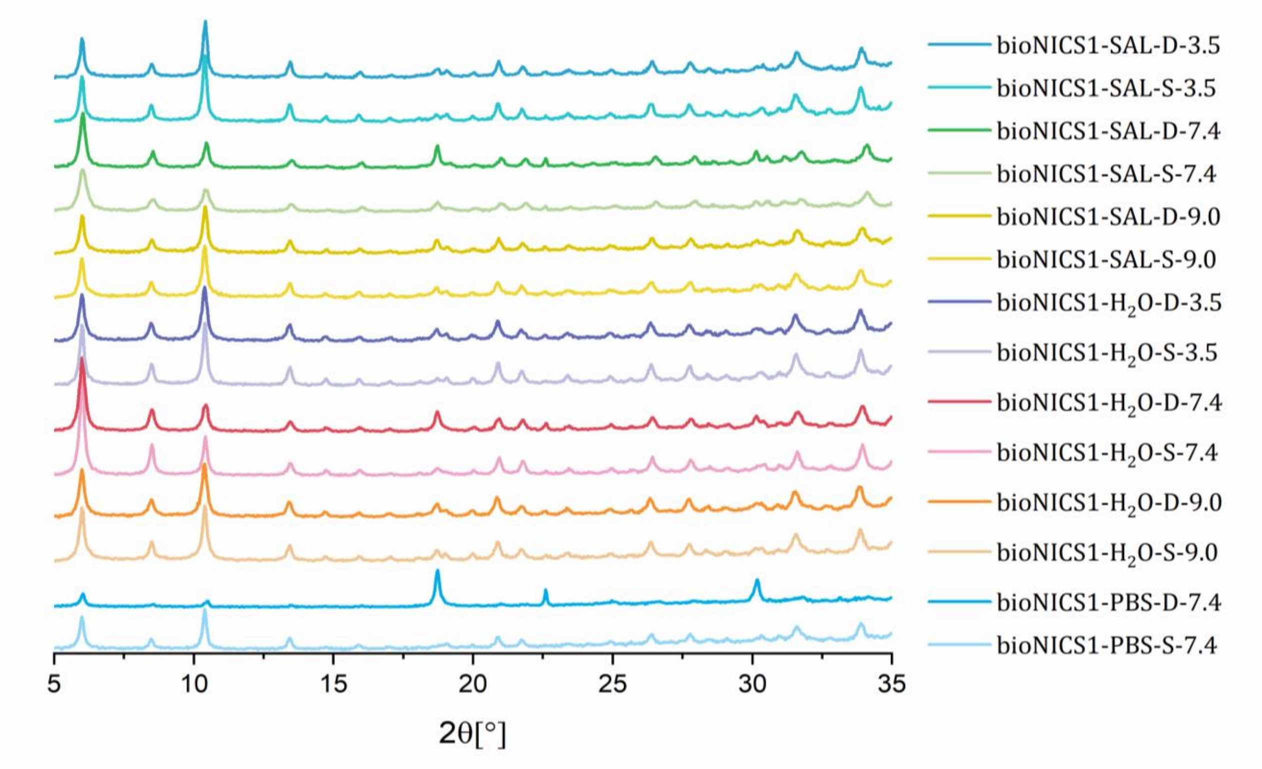

Supplementary Figure 12. XRPD patterns of the bioNICS-1 products treated under different aqueous conditions. Name indicates treatment conditions: bioNICS-1-x-y-z (x – sample immersed in demineralized water (H2O), saline solution (SAL) or phosphate buffer solution (PBS); y – sample left undisturbed (S) or under stirring (D); z – solution pH values).

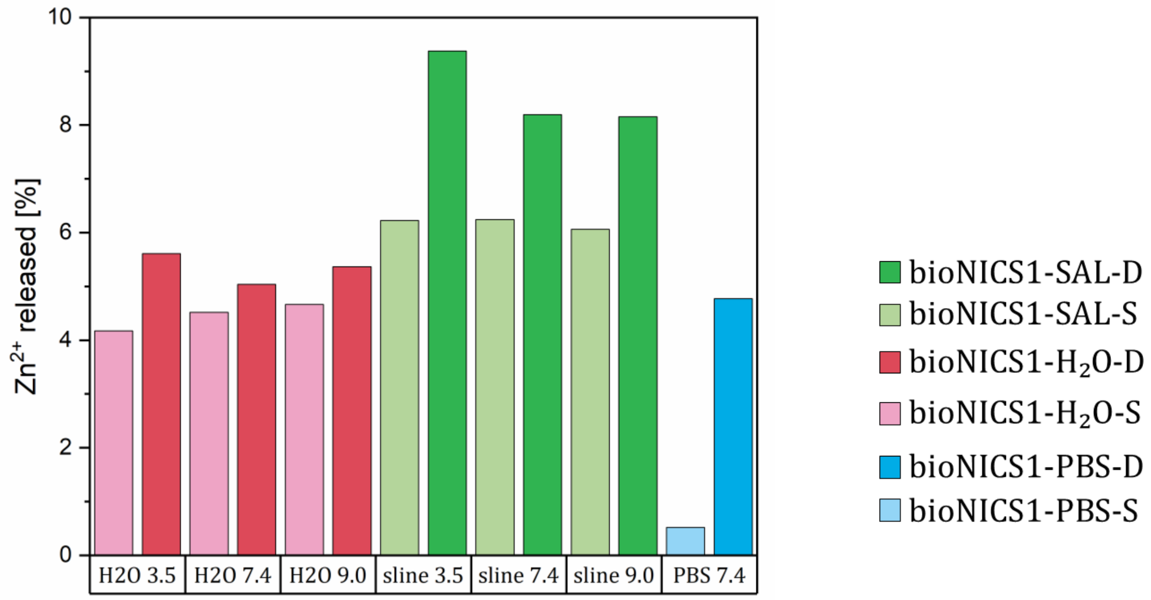

Supplementary Figure 13. Released % of Zn(II) cations from the bioNICS-1 framework within the supernatant calculated on the basis of the Zn(II) concentration measurements by ICP-OAS. Represented graphs differ type of release media, pH value and static or dynamic conditions.

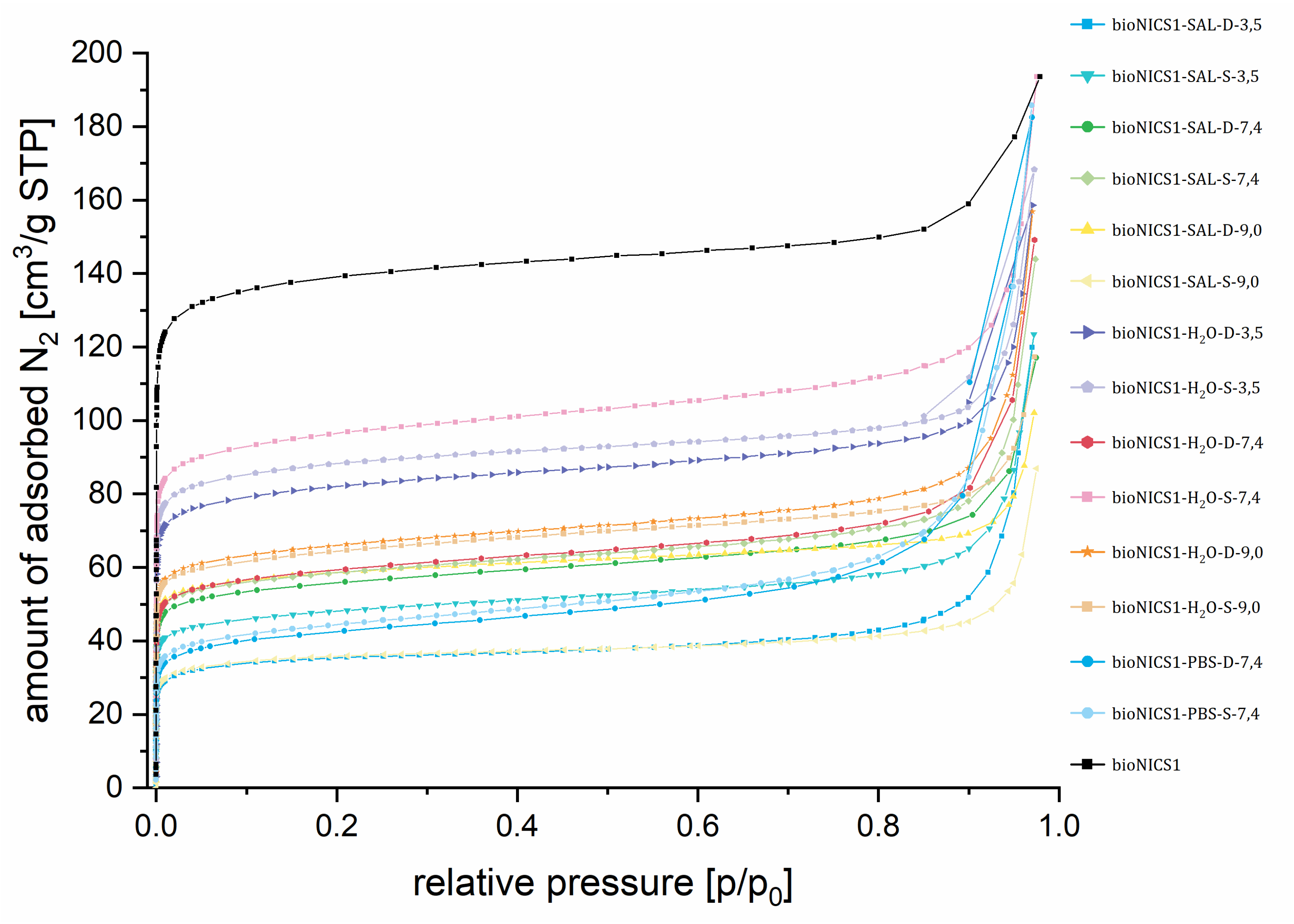

Supplementary Figure 14. N_2 isotherms of the bioNICS-1 products treated under different aqueous conditions. Name indicates treatment conditions: bioNICS-1-x-y-z (x – sample immersed in demineralized water (H_2O), saline solution (SAL) or phosphate buffer solution (PBS); y – sample left undisturbed (S) or under stirring (D); z – solution pH values). Hysteresis loops are omitted for clarity.

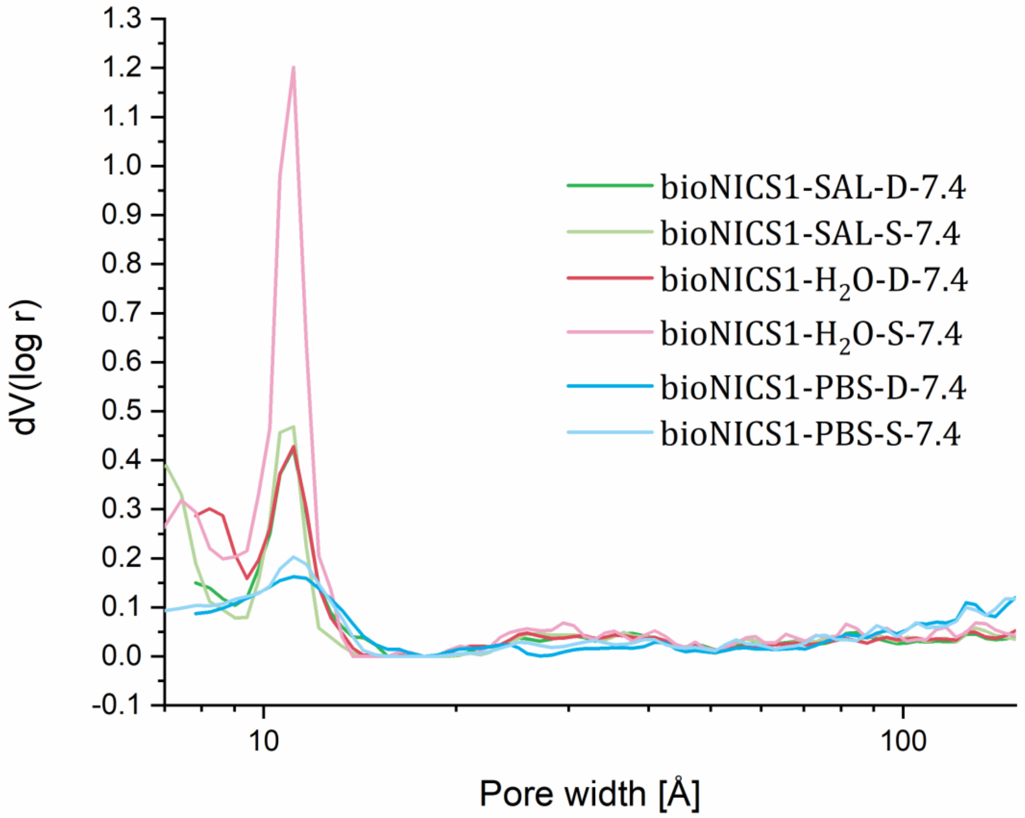

Supplementary Figure 15. Pore size distribution of bioNICS-1 products treated under different aqueous conditions. Name indicates treatment conditions: bioNICS-1-x-y-z (x – sample immersed in demineralized water (H_2O), saline solution (SAL) or phosphate buffer solution (PBS); y – sample left undisturbed (S) or under stirring (D); z – solution pH values).

Average pore size is not significantly affected by the dissolution media. However, pore size distribution becomes broader and micropore volume decreases in the case of exposure to PBS and saline solutions. The modification of micropores is strongly related to the BET surface area change and is caused either by enhanced solubility of bioNICS-1 in the case of saline solution or formation of additional insoluble nonporous phosphate-based shell domains in the case of PBS.

Supplementary Table S4. BET surface area and the remaining porosity relative to the parent material of the bioNICS-1 products treated under different aqueous conditions. Name indicates treatment conditions: bioNICS-1-x-y-z (x – sample immersed in demineralized water (H_2O), saline solution (SAL) or phosphate buffer solution (PBS); y – sample left undisturbed (S) or under stirring (D); z – solution pH values).

**sample**	**S_{BET} (m^2/g)**	**% BET recovered**
bioNICS-1-H_2O-D-7.4	228	41
bioNICS-1-SAL-D-7.4	211	38
bioNICS-1-PBS-D-7.4	160	28
bioNICS-1-H_2O-S-7.4	377	68
bioNICS-1-SAL-S-7.4	225	41
bioNICS-1-PBS-S-7.4	164	30
bioNICS-1-H_2O-S-9.0	249	45
bioNICS-1-H_2O-D-9.0	252	46
bioNICS-1-SAL-S-9.0	138	25
bioNICS-1-SAL-D-9.0	229	41
bioNICS-1-H_2O-S-3.5	346	63
bioNICS-1-H_2O-D-3.5	320	58
bioNICS-1-SAL-S-3.5	184	33
bioNICS-1-SAL-D-3.5	134	24

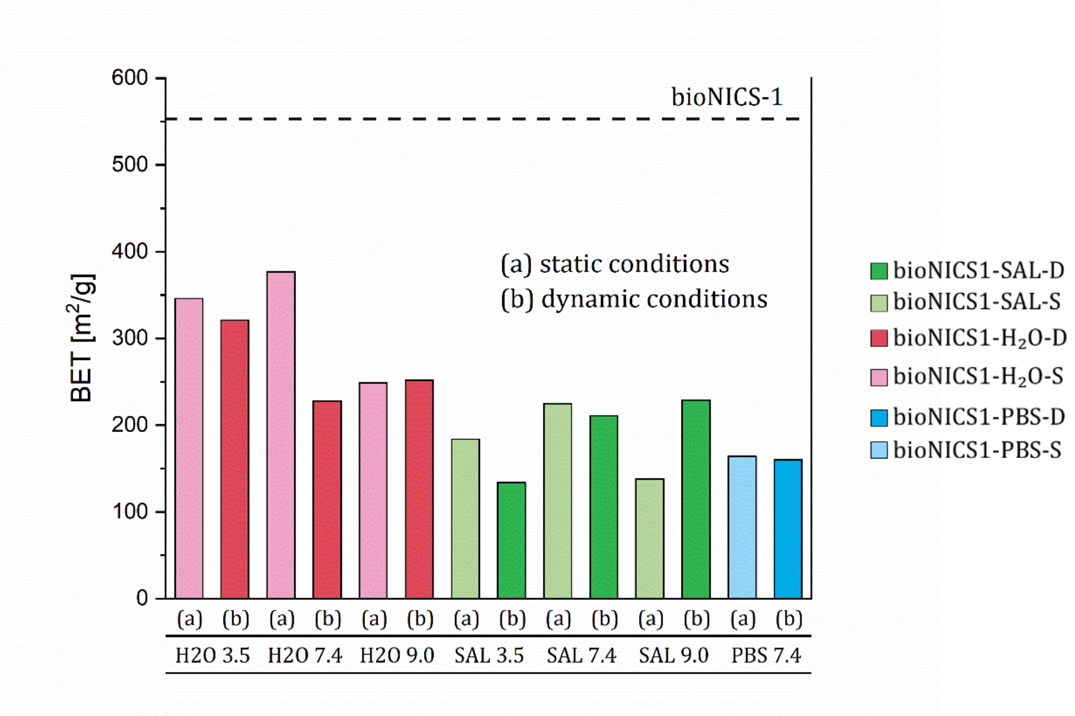

Supplementary Figure 16. BET specific surface area values of the solid products recovered after exposure in specified conditions. Dashed horizontal line indicates the BET value of the parent bioNICS-1 material.

2.2 Liquid NMR

The supernatant was investigated by ^{1}H liquid NMR spectroscopy to obtain information about release of the organic phase. The spectra confirm the release of organic material into the aqueous phase. The predominant species in most of the samples is characterized by peaks at δ_H 4.07 (1H, d, J=2.4 Hz), 4.02 (1H, ddd, J=7.8, 5.3, 2.4 Hz), 3.70 (1H, dd, J=11.5, 5.3 Hz) and 3.64 (1H, dd, J=11.5, 7.9 Hz). These do not conform to the expected chemical shifts of ascorbic acid, but match perfectly chemical shifts for threonic acid, a known degradation product of ascorbic acid, as reported in the human metabolome database[15]. This is further confirmed by ^{13}C, ^{1}H-^{13}C HSQC and ^{1}H-^{13}C HMBC NMR spectra, where we observed signals at four different ^{13}C NMR chemical shifts – δ_C 176.5, 70.7, 70.1 and 60.8 ppm for this compound. Threonic acid is the dominant species resulting from degradation of ascorbic acid in all but one sample, with concentrations of other degradation products roughly an order of magnitude lower according to integrals in ^{1}H NMR spectra. Additionally, signals from formic and acetic acids and for some samples also of ethanol, used in the preparation of the bioMOF material, are observed in the spectra.

Spectra of the two phosphate buffer solutions show a higher fraction of other degradation products of ascorbic acid, which we ascribe to the influence of phosphate ions on the stability and degradation pathways of ascorbic acid. The effect is even more pronounced for the sample resulting from treatment of the material in PBS under static conditions. The spectrum of this supernatant is complex, with signals present from a number of different compounds.

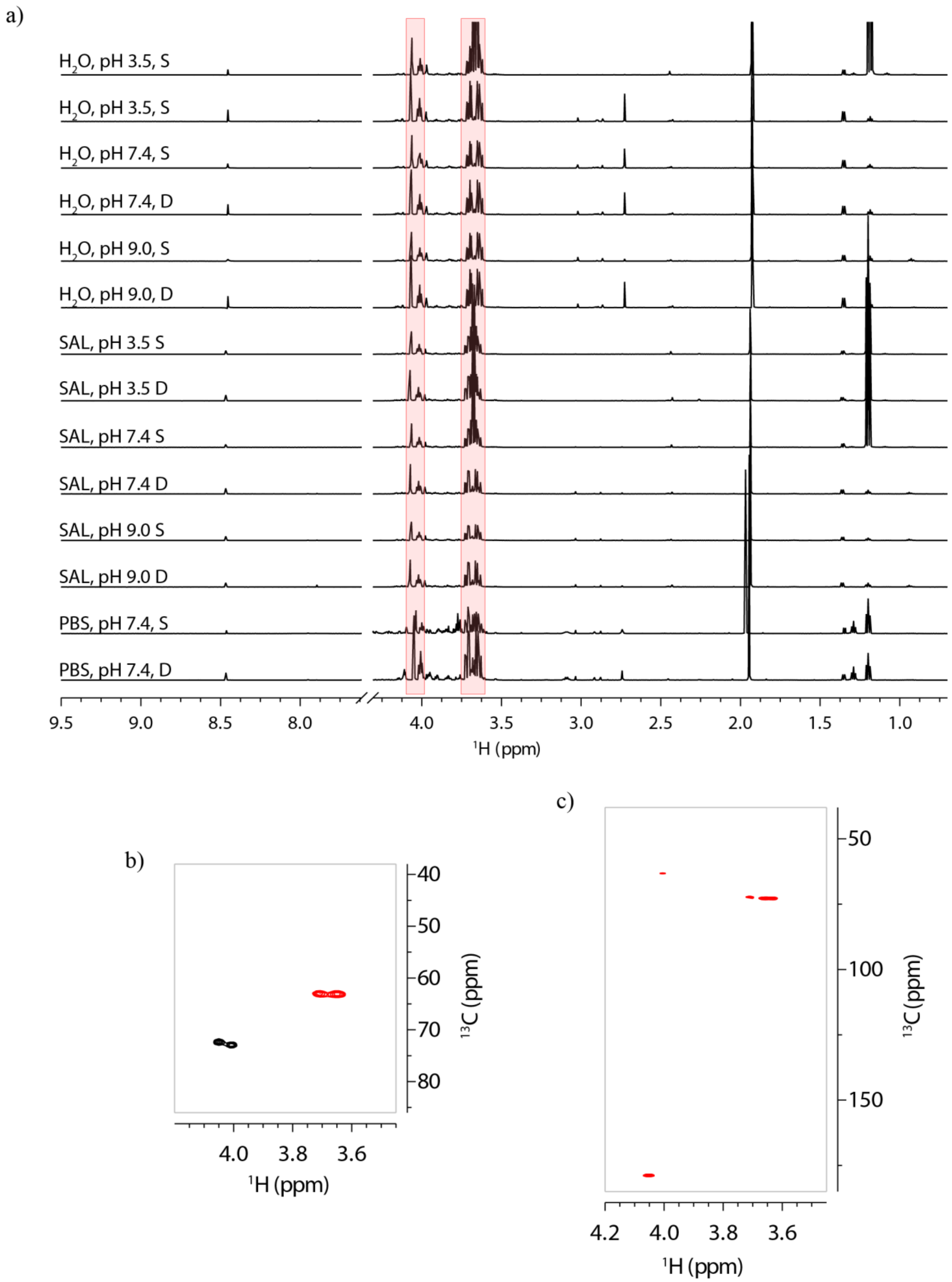

Supplementary Figure 17. (a) ^{1}H NMR spectra of the supernatants collected after treatment of bioNICS-1 in demineralised water (H_2O), saline solution (SAL) and phosphate buffer solution (PBS) at different pH values, left undisturbed (S) or with stirring (D). Regions containing signals matching the known spectral footprint of threonic acid are highlighted in the spectrum. Strips of ^{1}H-^{13}C HSQC (b) and HMBC (c) NMR spectra of the suprenatant after treatment of bioNICS-1 in PBS solution, pH 7.4, with stirring, showing the signals originating from the main degradation product of ascorbic acid.

3 Supplementary Note 2: Kinetics and mechanism of structure degradation

3.1 Zn(II) release kinetics.

Test was a kinetic modification of the previously described procedure presented by Howarth et al. Briefly, 250 mg of activated bioNICS-1 was suspended in 25 mL of 3 different media (purified water, saline and phosphate buffer solution), which represented about 5 mg of Zn / 1 mL media. Solutions had pH of 7.4 witch was regulated with HCl and/or NaOH. Samples of release medium (5 mL) were collected for analysis at set time intervals (1h, 2h, 4h, 6h, 12h, 24h, 48h, 72h, 96h and 168h) using a syringe with a 0.80 μm filter attached, and supplemented with the same volume of fresh media. This way the volume of the release medium stayed constant throughout the test, and *sink* conditions were maintained. The concentration of Zn(II) cations within the investigated solutions was determined by PerkinElmer AAnalyst 200 flame atomic absorption spectrometer (AAS).

Concentration of the released Zn(II) was calculated using Equation 1, where $C_{t(corr.)}$ is the corrected concentration at the time t, C_t is the apparent concentration at the time t, v is the volume of the sample taken and V is the total volume of the dissolution medium[16].

$$C_t(corr.) = C_t + \frac{v}{V}\sum_0^{t-1} C_t \qquad \text{(Eq. 1)}$$

3.2 Framework degradation kinetics in PBS.

85 mg of activated bioNICS-1 was suspended in 8,5 mL of PBS pH 7.4 and the immediately exposed to dynamic conditions on a circular plane mixer. At a set time point (5min, 10min, 20min, 40min, 1h, 2h, 4h, 8h, 12h, 24h, 48h, 72h, 96h) individual sample were filtered, dried on air over night and sediments identity checked with PXRD.

The relative crystallinity of the bioNICS-1 material recovered from PBS solution after different times of exposure was estimated by comparison of peak intensities corresponding to 011 reflection occurring at 6.0° 2θ. The weight contribution of Zn-oxalate which is formed during the above mentioned process was calculated from Rietveld quantification analysis using TOPAS Academic V6 software package[13].

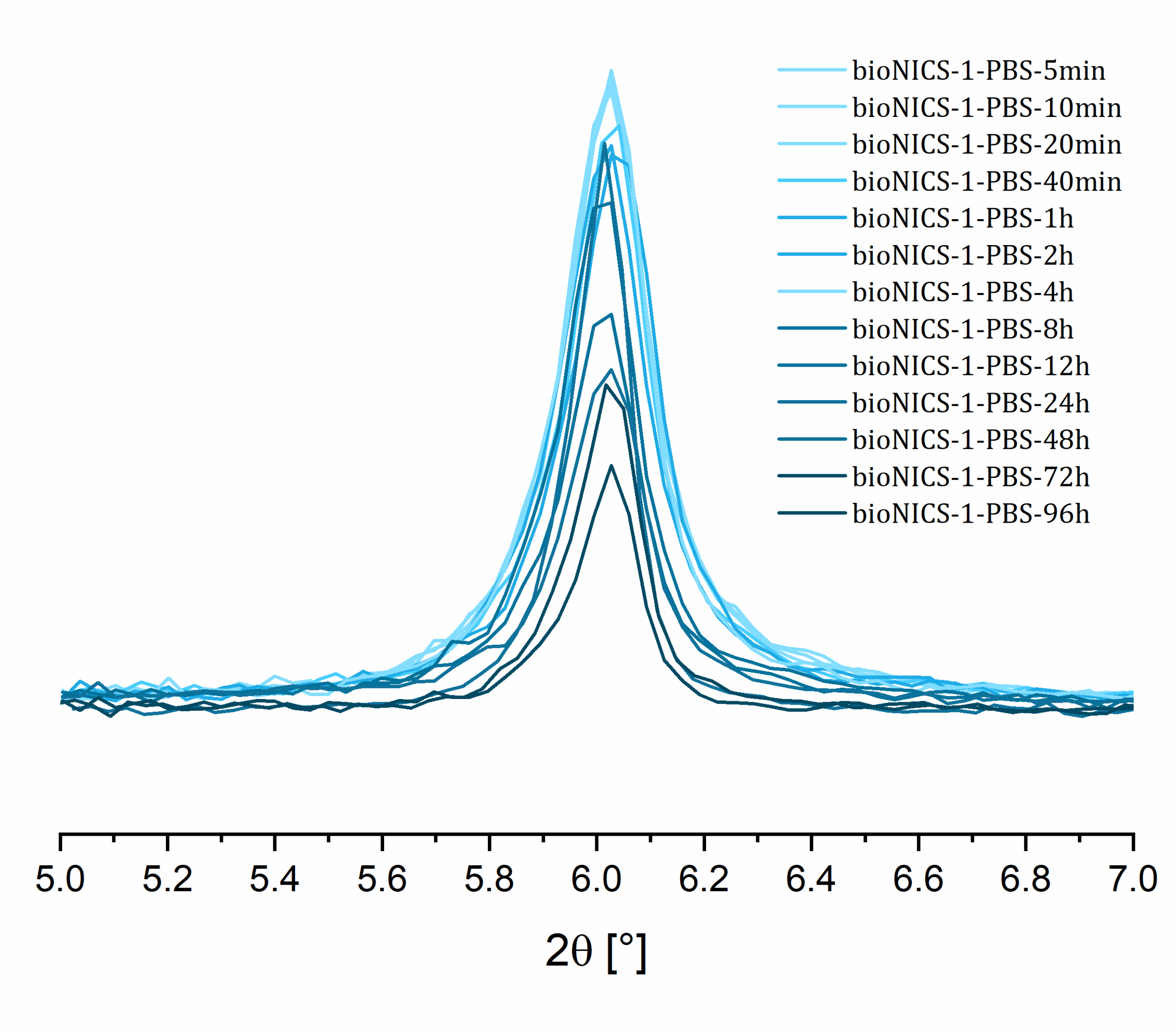

Supplementary Figure 18. XRPD patterns of the bioNICS-1 solids recovered from PBS solutions after stirring at specified times plotted in 2θ region between 5 and 7° indicating individual peak which corresponds to 011 reflection. The rate of crystallinity of bioNICS-1 framework structure was estimated according to the intensities of the 011 peak.

4 Supplementary Note 3: Impregnation of bioNICS-1 with a model drug - urea

Activated bioNICS-1 (200mg) was suspended in EtOH solution of urea (25 mg/mL) for 48h under constant stirring. Impregnation was done in two parallels, one at room temperature and one at 40°C. Both residues were filtered and washed with EtOH, then dried on air over night before proceeding with further tests.

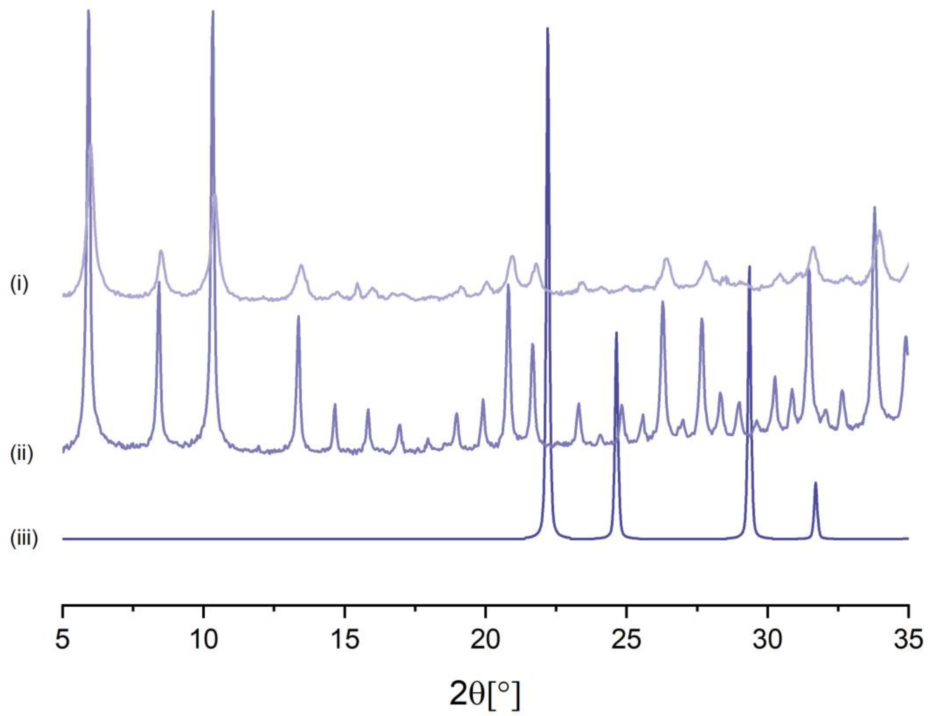

Supplementary Figure 19. XRD powder patterns of (i) bioNICS-1@urea, (ii) initial bioNICS-1 materials and (iii) referenced simulated urea pattern.

XRPD patterns evidence that the impregnation process did not alter the structure of the material and the absence of characteristic peaks of urea rules out the presence of free drug on the surface or elsewhere in bulk material. Urea content was estimated by TGA and liquid NMR and is in both cases approximately 10%. Additionally the reduction of BET surface confirms successful incorporation of guest molecules.

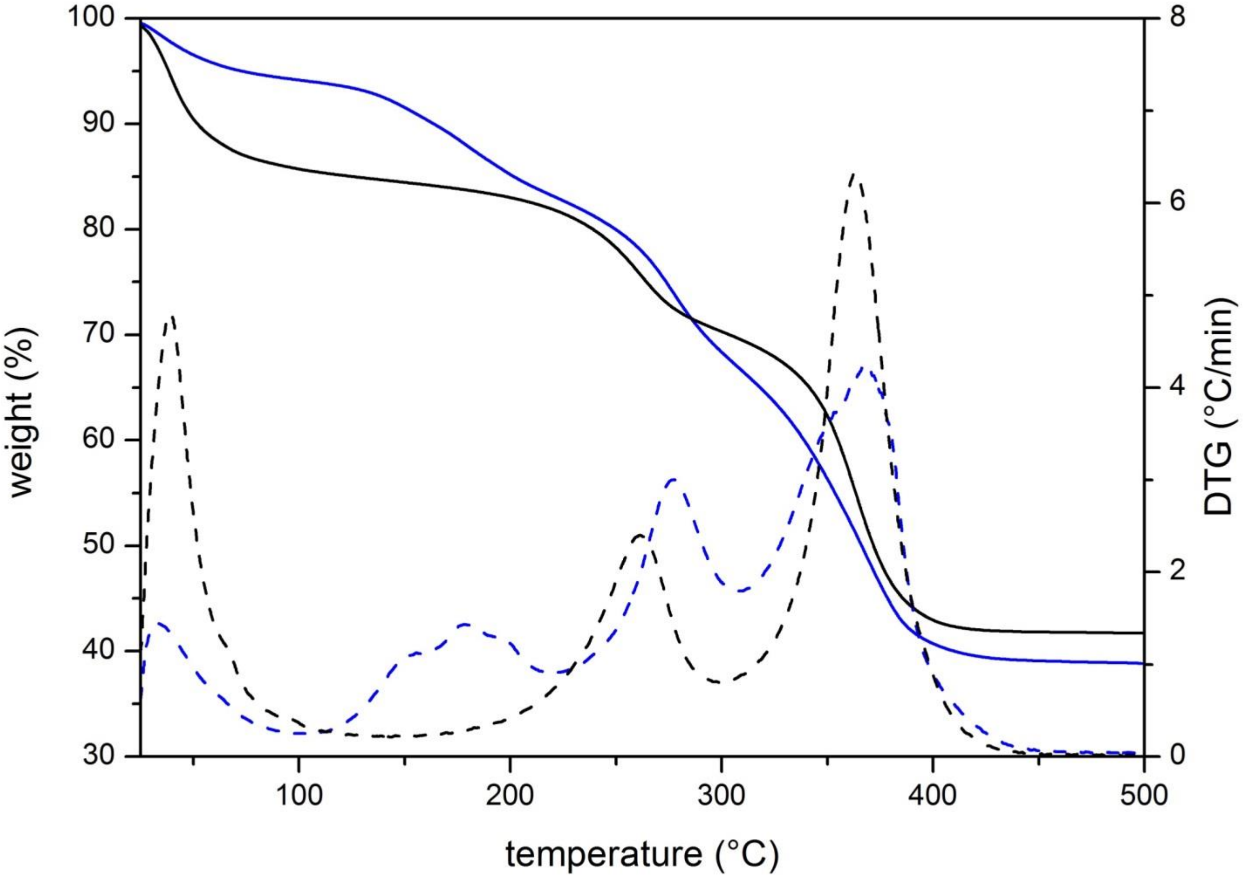

Supplementary Figure 20. TG/DTG curves of bioNICS-1@urea (blue) and initial bioNICS-1 (black) materials.

TGA showed an additional mass loss in the temperature range between 100 and 220 °C better observed from the DTG curve, which is an indication of the loaded molecule.

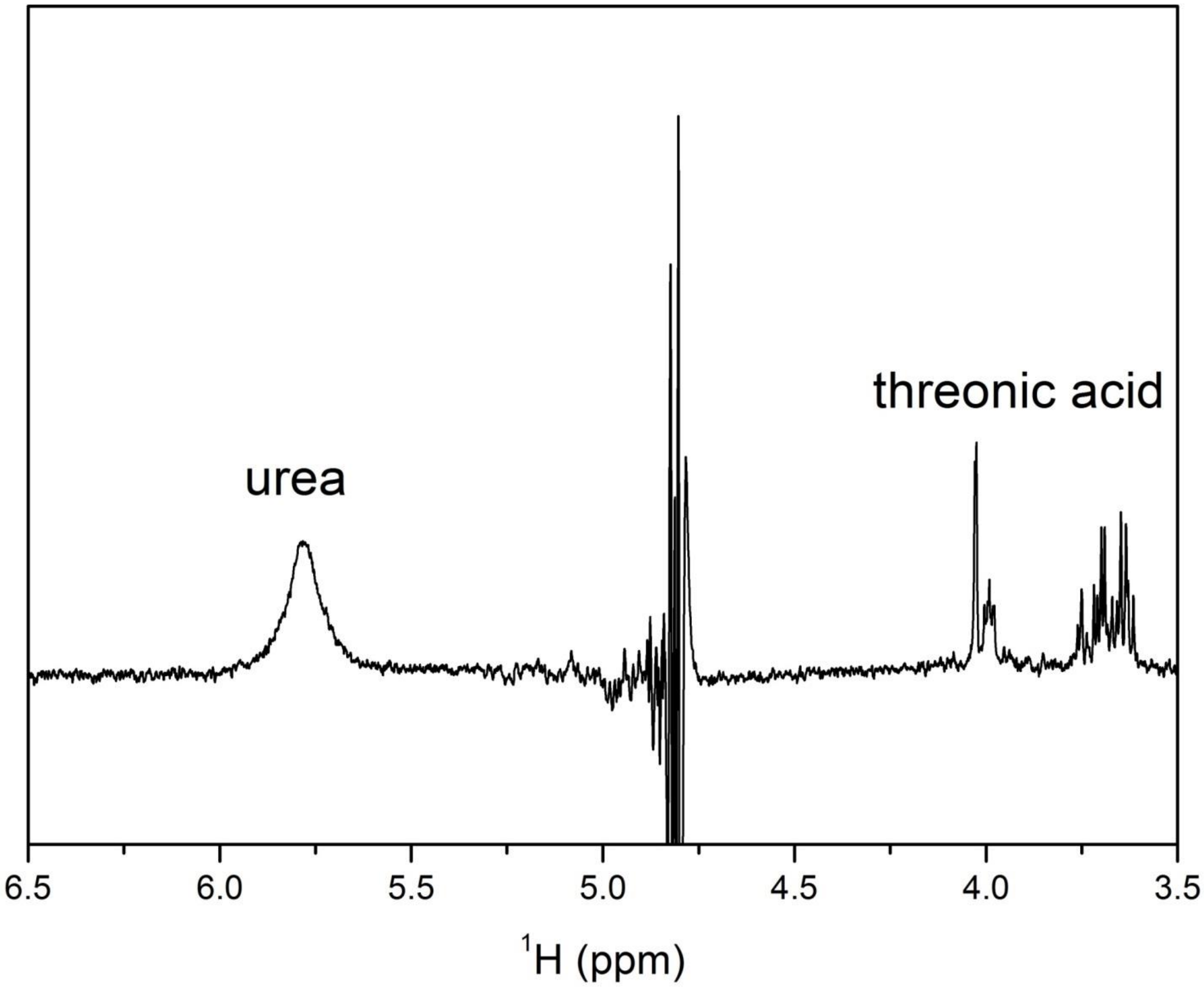

Supplementary Figure 21. Liquid ^{1}H NMR spectrum of dissolved bioNICS-1@urea in demineralized water. Spectrum was recorded with DPFGSE water suppression resulting in the noise at approximately 4.8 ppm.

^{1}H NMR spectrum showed characteristic shift belonging to urea. Further quantification determined that the concentration of loaded urea at 40 °C sample is 9 %. Spectrum indicates the additional presence of threonic acid in the solution which is a consequence of ascorbic acid degradation.

5 References

1. Zümreoglu-Karan, B. The coordination chemistry of Vitamin C: An overview. *Coordination Chemistry Reviews* **250**, 2295–2307 (2006).

2. Ferrer, E. G., Williams, P. A. M. & Baran, E. J. Interaction of the vanadyl(IV) cation with L-ascorbic acid and related systems. *Zeitschrift fur Naturforsch. - Sect. B J. Chem. Sci.* **53**, 256–262 (1998).

3. Starsich, F. H. L., Herrmann, I. K. & Pratsinis, S. E. Nanoparticles for Biomedicine: Coagulation During Synthesis and Applications. *https://doi.org/10.1146/annurev-chembioeng-060718-030203* **10**, 155–174 (2019).

4. Zahn, G. *et al.* A water-born Zr-based porous coordination polymer: Modulated synthesis of Zr-fumarate MOF. *Microporous Mesoporous Mater.* **203**, 186–194 (2015).

5. ISO/TS 80004-2:2015(en), Nanotechnologies — Vocabulary — Part 2: Nano-objects. Available at: https://www.iso.org/obp/ui/#iso:std:iso:ts:80004:-2:ed-1:v1:en. (Accessed: 8th June 2020)

6. Dadwal, A., Baldi, A. & Narang, R. K. Nanoparticles as carriers for drug delivery in cancer. *https://doi.org/10.1080/21691401.2018.1457039* **46**, 295–305 (2018).

7. Gaumet, M., Vargas, A., Gurny, R. & Delie, F. Nanoparticles for drug delivery: The need for precision in reporting particle size parameters. *European Journal of Pharmaceutics and Biopharmaceutics* **69**, 1–9 (2008).

8. Cichocka, M. O. *et al.* High-throughput continuous rotation electron diffraction data acquisition via software automation. *urn:issn:1600-5767* **51**, 1652–1661 (2018).

9. Kabsch, W. & IUCr. XDS. *urn:issn:0907-4449* **66**, 125–132 (2010).

10. Sheldrick, G. M. & IUCr. SHELXT – Integrated space-group and crystal-structure determination. *urn:issn:2053-2733* **71**, 3–8 (2015).

11. Sheldrick, G. M. & IUCr. A short history of SHELX. *urn:issn:0108-7673* **64**, 112–122 (2007).

12. Burla, M. C. *et al.* Crystal structure determination and refinement via SIR2014. *urn:issn:1600-5767* **48**, 306–309 (2015).

13. Coelho, A. A. TOPAS-Academic V6. Coelho Software (2016).

14. Howarth, A. J. *et al.* Best Practices for the Synthesis, Activation, and Characterization of Metal–Organic Frameworks. *Chem. Mater.* **29**, 26–39 (2017).

15. Englard, S. & Seifter, S. The Biochemical Functions of Ascorbic Acid. *Annu. Rev. Nutr.*

6, 365–406 (1986).

16. Fisher, K. A., Huddersman, K. D. & Taylor, M. J. Comparison of Micro- and Mesoporous Inorganic Materials in the Uptake and Release of the Drug Model Fluorescein and Its Analogues. *Chem. - A Eur. J.* **9**, 5873–5878 (2003).
